# High-Electrical-Conductivity Multilayer Graphene Formed by Layer Exchange with Controlled Thickness and Interlayer

**DOI:** 10.1038/s41598-019-40547-0

**Published:** 2019-03-11

**Authors:** Hiromasa Murata, Yoshiki Nakajima, Noriyuki Saitoh, Noriko Yoshizawa, Takashi Suemasu, Kaoru Toko

**Affiliations:** 1 Institute of Applied Physics, University of Tsukuba, 1-1-1 Tennodai, Tsukuba, Ibaraki 305-8573 Japan; 2 Electron Microscope Facility, TIA, AIST, 16-1 Onogawa, Tsukuba, 305-8569 Japan; 3 PRESTO, Japan Science and Technology Agency, 4-1-8 Honcho, Kawaguchi, Saitama 332-0012 Japan

## Abstract

The layer exchange technique enables high-quality multilayer graphene (MLG) on arbitrary substrates, which is a key to combining advanced electronic devices with carbon materials. We synthesize uniform MLG layers of various thicknesses, *t*, ranging from 5 nm to 200 nm using Ni-induced layer exchange at 800 °C. Raman and transmission electron microscopy studies show the crystal quality of MLG is relatively low for *t* ≤ 20 nm and dramatically improves for *t* ≥ 50 nm when we prepare a diffusion controlling Al_2_O_3_ interlayer between the C and Ni layers. Hall effect measurements reveal the carrier mobility for *t* = 50 nm is 550 cm^2^/Vs, which is the highest Hall mobility in MLG directly formed on an insulator. The electrical conductivity (2700 S/cm) also exceeds a highly oriented pyrolytic graphite synthesized at 3000 °C or higher. Synthesis technology of MLG with a wide range of thicknesses will enable exploration of extensive device applications of carbon materials.

## Introduction

Multilayer graphene (MLG) has excellent characteristics, such as high electrical/thermal conductivities and current-carrying capacity exceeding that of Cu^1–4^. Therefore, application of MLG on arbitrary substrates is expected in various applications, including transparent electrodes, low-resistance wiring, and heat spreaders. As the required MLG thickness depends on the application, a technique for controlling the thickness of high-quality MLG film is essential.

High-quality graphene and MLG have been produced on arbitrary substrates using transfer techniques^5^ and chemical vapor deposition^6–11^. Some of these techniques can precisely control the numbers of graphene layers; however, there is difficulty forming thick MLG in the tens of nanometers. Against this backdrop, metal-induced solid-phase crystallization of amorphous carbon (a-C) or polymers has attracted increased attention owing to the direct synthesis of MLG on insulators^12–26^. Some of these techniques have allowed synthesis of thick (>5 nm) MLG by controlling the initial thickness of a-C^17–26^. However, a problem has existed with the uniformity of the MLG layer, which makes it difficult to systematically evaluate the electrical properties of such layers.

Metal-induced layer exchange (MILE) has been actively studied in the field of group-IV semiconductors, including Si^27–31^, Ge^32–37^, and SiGe^38,39^. In MILE, an amorphous semiconductor layer crystallizes through “layer exchange” between the amorphous layer and a catalyst metal layer. The thickness design of the initial metal layer can easily control the resulting semiconductor layer^27,31,35^. We found the layer exchange occurs in the Co-C and Ni-C systems and fabricated a uniform 50-nm-thick MLG on an insulator^40,41^. Furthermore, we employed a diffusion controlling interlayer (IL) between C and Ni, which suppresses the nucleation of the MLG and results in enlargement of MLG grains^42^. In MILE, the initial metal thickness strongly influences the crystallinity of the resulting semiconductor layer^31,38^. This study first clarifies the effect of film thickness on the crystallinity and electrical properties of MLG on an insulator. The result shows a carrier mobility of 550 cm^2^/Vs and an electrical conductivity of 2700 S/cm; the highest values among most MLG layers directly formed on insulators.

## Results

**Figure 1:**
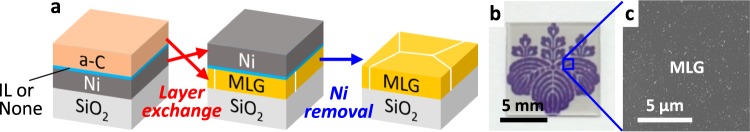
Typical example of layer exchange. (**a**) Schematic of the sample preparation procedure. (**b**) Photograph and (**c**) SEM image of the sample for *t* = 5 nm without IL after Ni removal.

As shown in Fig. 1a, all samples started with a stacked structure of an a-C/Ni/SiO_2_ substrate and were then annealed at 800 °C for 1 h in ambient Ar. We prepared the samples with and without a diffusion controlling Al_2_O_3_ IL, which improves the crystal quality of MLG^42^. The thickness of the initial Ni layer determines the resulting MLG thickness, *t*, after layer exchange^41,42^. We varied *t* from 5 nm to 200 nm to investigate the effects of *t* on the crystal and electrical properties of the MLG. For all samples, MLG layers were obtained on the substrate via layer exchange. As representatively shown in Fig. 1b, thin MLG layers (*t* ≤ 10 nm) exhibited transparency. The scanning electron microscopy (SEM) image in Fig. 1c shows that although the MLG layer has submicron-size voids, it covers nearly the entire substrate. We note the Ni concentration in the MLG layer is below the detection limit of EDX (~1%).

**Figure 2:**
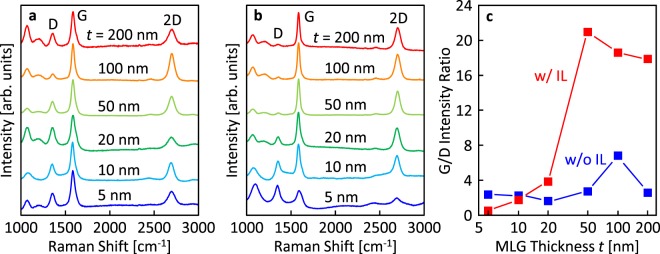
Raman study of MLG formed by layer exchange. Raman spectra obtained from back side of the samples before Ni removal (**a**) without and (**b**) with the IL. (**c**) G/D intensity ratio of the samples determined by the Raman spectra shown in (**a**,**b**), as a function of *t*.

Figure 2a,b show that Raman spectra of the back side of the samples have peaks at approximately 1350, 1580, and 2700 cm^−1^, corresponding to the D (disordered mode), G (graphitic mode), and 2D (D mode overtone) peaks in the graphitic structure, respectively. This means layer exchange between the C and Ni layers occurred and MLG formed on the SiO_2_ glass substrate in all samples. In the Raman spectra, the intensity ratio of the G to D peaks (*I*_G_/*I*_D_) in the spectra corresponds to the crystal quality of MLG^43^. Figure 2c indicates the *I*_G_/*I*_D_ ratio strongly depends on *t*, and with or without the IL. For the samples without the IL, the *I*_G_/*I*_D_ ratio shows the highest value of 7 at *t* = 100 nm. For the samples with the IL, the *I*_G_/*I*_D_ ratio is as low as for the samples without the IL for *t* ≤ 20 nm, while it dramatically increases for *t* ≥ 50 nm and exceeds 20. The crystal quality of the MLG, estimated from the *I*_G_/*I*_D_ ratio, likely reflects the contamination from the substrate and the grain size enlargement by inserting the IL^42^. Although the *I*_G_/*I*_D_ ratio of 20 is still lower than that of MLG formed by the transfer technique or high-temperature CVD^4^, this value is the highest among MLG synthesized on insulators by metal-induced solid-phase crystallization^18,19,23,25^.

**Figure 3:**
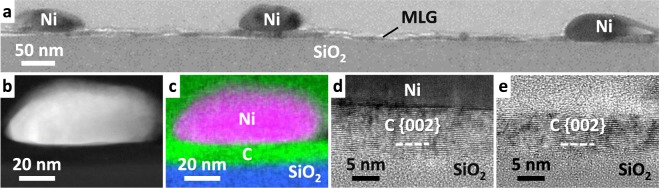
Characterization of the cross-section of the sample for *t* = 10 nm without the IL before Ni removal. (**a**) Bright-field TEM image. (**b**) HAADF-STEM image and (**c**) EDX elemental map of an Ni-contacted region. High-resolution lattice images of the MLG layer showing (**d**) an Ni-contacted region and (**e**) a non-Ni-contacted region.

The detailed cross-sectional structure of the sample for *t* = 10 nm, which is a typical thin transparent film, was investigated using an analytical transmission electron microscope (TEM). Figure 3a shows the MLG formed on the entire substrate. Figure 3b,c show Ni on the MLG layer is particle-shaped, whereas the sample with *t* = 50 nm has a continuous Ni layer^41,42^. Considering that the MLG layer is uniformly formed, the Ni particles are likely attributed to the agglomeration of a thin Ni layer moved on MLG after layer exchange. Figure 3d,e show {002} oriented MLG forms on the SiO_2_ substrate in both the Ni-contacted and non-Ni-contacted regions. The MLG in the non-Ni-contacted region is slightly thinner than that in the Ni-contacted region, probably because of the C evaporation during annealing. Figure 3d,e indicate the crystal structure of the thin MLG is disordered compared with the sample with an IL^42^. We note that, for *t* = 50 nm, the grain size of the MLG layer without an IL was a few hundred nm and that with an IL was a few μm^41,42^. These results account well for the Raman study (Fig. 2c). For high-quality MLG, such as that synthesized by thermal decomposition of a SiC substrate, TEM observation can identify the stacking structure (e.g. AA and AB stacking)^44,45^. Unfortunately, because the current MLG is small-grained polycrystalline where the grains are randomly oriented in the in-plane direction^42^, it is difficult to identify the stacking.

**Figure 4:**
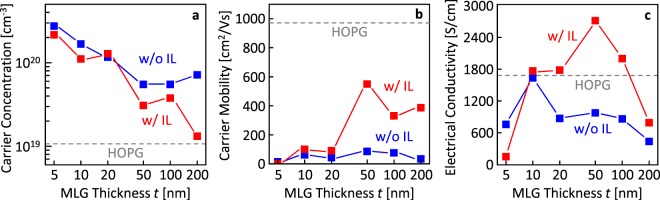
Electrical properties of the MLG layers after Ni removal as a function of *t*. (**a**) Carrier concentration. (**b**) Carrier mobility. (**c**) Electrical conductivity. The data of a HOPG are shown as dotted lines.

Figure 4 shows the electrical properties of the MLG strongly depend on *t*, and with or without the IL. Figure 4a shows the carrier concentration of the MLG decreases with the increase of *t* and approaches the value of a highly oriented pyrolytic graphite (HOPG) with a low mosaic degree of 0.4°, especially for the sample with the IL. Figure 4b shows the carrier mobility dramatically increases for the *t* ≥ 50 nm samples with the IL, whereas it remains low in the whole range of *t* for the samples without the IL. The carrier mobility exhibits the maximum value of 550 cm^2^/Vs for the *t* = 50 nm sample with the IL, which is the highest Hall mobility among MLGs directly formed on an insulator. Figure 4c shows the behavior of the electrical conductivity reflects that of the carrier concentration and carrier mobility. The electrical conductivity exhibits the maximum value of 2700 S/cm for the *t* = 50 nm sample with the IL, which exceeds that of the HOPG synthesized at 3000 °C or higher.

## Discussion

We have controlled the thickness of MLG on an insulator formed by layer exchange with and without the IL, resulting in a high electrical conductivity. Considering the fact that the behavior of the carrier mobility (Fig. 4b) is the same as that of the *I*_G_/*I*_D_ ratio in the Raman spectra (Fig. 2c), the carrier mobility clearly reflects the crystal quality of the MLG. The carrier mobility, that is, the crystal quality of the MLG is much higher than any other MLG directly formed on an insulator; however, does not reach the HOPG. The electrical conductivity of the MLG exceeds that of HOPG with the aid of autodoping. Although the reasons for the excessive carriers for the MLG are still unclear, when we assume defects in MLG produce carriers, the carrier concentration behavior is almost consistent with the Raman study.

In conclusion, the crystal quality and electrical properties of the MLG, formed on an insulator by layer exchange at 800 °C, strongly depended on *t*, especially when preparing the IL. The crystal quality was relatively low for *t* ≤ 20 nm; however, it dramatically improved for *t* ≥ 50 nm with the IL. As a result, the sample for *t* = 50 nm with the IL exhibited the maximum carrier mobility (550 cm^2^/Vs), and the highest Hall mobility among MLG directly formed on an insulator. The electrical conductivity (2700 S/cm) even exceeded the high-quality HOPG (mosaic degree: 0.4°). Thus, the layer exchange method allows us to form MLG with a wide range of thicknesses on arbitrary substrates. This opens the door for a broad range of applications that combine advanced electronic devices with carbon materials.

## Methods

### Sample preparation

Ni thin films with thicknesses of 5, 10, 20, 50, 100, and 200 nm were prepared on SiO_2_ glass substrates. Subsequently, Al_2_O_3_ (thickness: 2 nm) was employed as the diffusion control IL^42^. Then, a-C thin films were prepared, wherein the thickness ratio of C:Ni = 3:2 (e.g., C:Ni = 300:200 nm). All depositions were carried out using radio-frequency (RF) magnetron sputtering (base pressure: 3.0 × 10^−4^ Pa) with an Ar plasma. During the deposition, the substrate temperature was room temperature for Al_2_O_3_ and 200 °C for Ni and C. The RF power was set to 100 W for C and 50 W for Ni and Al_2_O_3_. For comparison, we prepared the samples without the Al_2_O_3_ IL. Samples were annealed at 800 °C for 1 h in an Ar ambient to induce layer exchange growth. The sample was dipped in a diluted HNO_3_ solution (10% HNO_3_) for 30 min to remove Ni moved to the top layer.

### Material characterization

SEM analyses were performed using a JEOL JSM-7001F with an EDX spectrometer (JEOL JEO-2300). Raman spectroscopy was performed using a JASCO NRS-5100, wherein the laser wavelength was 532 nm and the spot size was 5 µm. TEM analyses were performed using an analytical TEM, FEI Tecnai Osiris, operating at 200 kV, equipped with an EDX spectrometer (FEI Super-X system). The cross-sectional TEM sample was prepared using the conventional focused ion beam method. Hall effect measurement was performed with the Van der Pauw method using the Bio-Rad HL5500PC system. Carrier mobility, carrier concentration, and electrical conductivity were averaged over five measurements for each sample.

## Data Availability

The data used in this study are available upon reasonable request from the corresponding author K.T. (toko@bk.tsukuba.ac.jp).

## References

[1] Biswas S, Drzal LT (2010). Multilayered nano-architecture of variable sized graphene nanosheets for enhanced supercapacitor electrode performance. ACS Appl. Mater. Interfaces.

[2] Balandin AA (2011). Thermal properties of graphene and nanostructured carbon materials. Nat. Mater..

[3] Murali R, Yang Y, Brenner K, Beck T, Meindl JD (2009). Breakdown current density of graphene nanoribbons. Appl. Phys. Lett..

[4] Kim KS (2009). Large-scale pattern growth of graphene films for stretchable transparent electrodes. Nature.

[5] Novoselov KS (2004). Electric field effect in atomically thin carbon films. Science.

[6] Su C-Y (2011). Direct formation of wafer scale graphene thin layers on insulating substrates by chemical vapor deposition. Nano Lett..

[7] Kato T, Hatakeyama R (2012). Direct growth of doping-density-controlled hexagonal graphene on SiO_2_ substrate by rapid-heating plasma CVD. ACS Nano.

[8] Yen W-CC (2014). Direct growth of self-crystallized graphene and graphite nanoballs with Ni vapor-assisted growth: from controllable growth to material characterization. Sci. Rep..

[9] Murakami K (2015). Direct synthesis of large area graphene on insulating substrate by gallium vapor-assisted chemical vapor deposition. Appl. Phys. Lett..

[10] Chugh S (2015). Comparison of graphene growth on arbitrary non-catalytic substrates using low-temperature PECVD. Carbon.

[11] Ueno K, Ichikawa H, Uchida T (2016). Effect of current stress during thermal CVD of multilayer graphene on cobalt catalytic layer. Jpn. J. Appl. Phys..

[12] Peng Z, Yan Z, Sun Z, Tour JM (2011). Direct growth of bilayer graphene on SiO_2_ substrates by carbon diffusion through nickel. ACS Nano.

[13] Kwak J (2012). Near room-temperature synthesis of transfer-free graphene films. Nat. Commun..

[14] Banno K (2013). Transfer-free graphene synthesis on insulating substrates via agglomeration phenomena of catalytic nickel films. Appl. Phys. Lett..

[15] Xiong W (2015). Solid-state graphene formation via a nickel carbide intermediate phase. RSC Adv..

[16] Berman D (2016). Metal-induced rapid transformation of diamond into single and multilayer graphene on wafer scale. Nat. Commun..

[17] Vishwakarma R (2017). Transfer free graphene growth on SiO_2_ substrate at 250 °C. *Sci*. Rep..

[18] Byun SJ (2011). Graphenes converted from polymers. J. Phys. Chem. Lett..

[19] Gumi K, Ohno Y, Maehashi K, Inoue K, Matsumoto K (2012). Direct Synthesis of Graphene on SiO_2_ Substrates by Transfer-Free Processes. Jpn. J. Appl. Phys..

[20] Weatherup RS (2013). Introducing carbon diffusion barriers for uniform, high-quality graphene growth from solid sources. Nano Lett..

[21] Tamaoki M, Imaeda H, Kishimoto S, Mizutani T (2013). Transfer-free fabrication of graphene field effect transistor arrays using solid-phase growth of graphene on a SiO_2_/Si substrate. Appl. Phys. Lett..

[22] Tanaka H, Obata S, Saiki K (2013). Reduction of graphene oxide at the interface between a Ni layer and a SiO_2_ substrate. Carbon.

[23] Sato M (2014). Intercalated multilayer graphene wires and metal/multilayer graphene hybrid wires obtained by annealing sputtered amorphous carbon. Jpn. J. Appl. Phys..

[24] Kosaka M, Takano S, Hasegawa K, Noda S (2015). Direct synthesis of few- and multi-layer graphene films on dielectric substrates by “etching-precipitation” method. Carbon.

[25] Zhuo QQ (2015). Transfer-free synthesis of doped and patterned graphene films. ACS Nano.

[26] Yamada J, Ueda Y, Maruyama T, Naritsuka S (2016). Direct growth of multilayer graphene by precipitation using W capping layer. Jpn. J. Appl. Phys..

[27] Nast O, Puzzer T, Koschier LM, Sproul AB, Wenham SR (1998). Aluminum-induced crystallization of amorphous silicon on glass substrates above and below the eutectic temperature. Appl. Phys. Lett..

[28] Sarikov A (2010). A kinetic simulation study of the mechanisms of aluminum induced layer exchange process. J. Appl. Phys..

[29] Wang Z, Gu L, Jeurgens LPH, Phillipp F, Mittemeijer EJ (2012). Real-time visualization of convective transportation of solid materials at nanoscale. Nano Lett..

[30] Birajdar BI, Antesberger T, Butz B, Stutzmann M, Spiecker E (2012). Direct *in situ* transmission electron microscopy observation of Al push up during early stages of the Al-induced layer exchange. Scr. Mater..

[31] Toko K (2014). Selective formation of large-grained, (100)- or (111)-oriented Si on glass by Al-induced layer exchange. J. Appl. Phys..

[32] Toko K (2012). Highly (111)-oriented Ge thin films on insulators formed by Al-induced crystallization. Appl. Phys. Lett..

[33] Park J-H, Kasahara K, Hamaya K, Miyao M, Sadoh T (2014). High carrier mobility in orientation-controlled large-grain (≥50 μm) Ge directly formed on flexible plastic by nucleation-controlled gold-induced-crystallization. Appl. Phys. Lett..

[34] Toko K (2014). Low-temperature (180 °C) formation of large-grained Ge (111) thin film on insulator using accelerated metal-induced crystallization. Appl. Phys. Lett..

[35] Toko K, Nakazawa K, Saitoh N, Yoshizawa N, Suemasu T (2015). Improved surface quality of the metal-induced crystallized Ge seed layer and its influence on subsequent epitaxy. Cryst. Growth Des..

[36] Higashi H (2018). Electrical properties of pseudo-single-crystalline Ge films grown by Au-induced layer exchange crystallization at 250 °C. J. Appl. Phys..

[37] Yoshimine R, Toko K, Saitoh N, Yoshizawa N, Suemasu T (2017). Silver-induced layer exchange for polycrystalline germanium on a flexible plastic substrate. J. Appl. Phys..

[38] Kurosawa M, Kawabata N, Sadoh T, Miyao M (2012). Enhanced interfacial-nucleation in Al-induced crystallization for (111) oriented Si_1–*x*_Ge_*x*_ (0 ≤ x ≤ 1) films on insulating substrates. ECS J. Solid State Sci. Technol..

[39] Toko K, Kusano K, Nakata M, Suemasu T (2017). Low temperature synthesis of highly oriented p-type Si_1−*x*_Ge_*x*_ (*x*: 0–1) on an insulator by Al-induced layer exchange. J. Appl. Phys..

[40] Murata H, Toko K, Suemasu T (2017). Multilayer graphene on insulator formed by Co-induced layer exchange. Jpn. J. Appl. Phys..

[41] Murata H, Toko K, Saitoh N, Yoshizawa N, Suemasu T (2017). Direct synthesis of multilayer graphene on an insulator by Ni-induced layer exchange growth of amorphous carbon. Appl. Phys. Lett..

[42] Murata H, Saitoh N, Yoshizawa N, Suemasu T, Toko K (2017). High-quality multilayer graphene on an insulator formed by diffusion controlled Ni-induced layer exchange. Appl. Phys. Lett..

[43] Chu PK, Li L (2006). Characterization of amorphous and nanocrystalline carbon films. Mater. Chem. Phys..

[44] Borysiuk J (2012). Role of structure of C-terminated 4H-SiC(000-1) surface in growth of graphene layers: Transmission electron microscopy and density functional theory studies. Phys. Rev. B..

[45] Borysiuk J (2014). Structural defects in epitaxial graphene layers synthesized on C-terminated 4H-SiC (000-1) surface-Transmission electron microscopy and density functional theory studies. J. Appl. Phys..

